# Cerebrospinal Fluid and Serum Biomarker Insights in Aneurysmal Subarachnoid Haemorrhage: Navigating the Brain–Heart Interrelationship for Improved Patient Outcomes

**DOI:** 10.3390/biomedicines11102835

**Published:** 2023-10-19

**Authors:** Małgorzata Burzyńska, Agnieszka Uryga, Rafał Załuski, Anna Goździk, Barbara Adamik, Chiara Robba, Waldemar Goździk

**Affiliations:** 1 Clinical Department of Anaesthesiology and Intensive Care, Wroclaw Medical University, 50-367 Wroclaw, Poland; malgorzata.burzynska@umw.edu.pl (M.B.); waldemar.gozdzik@umw.edu.pl (W.G.); 2 Department of Biomedical Engineering, Faculty of Fundamental Problems of Technology, Wroclaw University of Science and Technology, 50-370 Wroclaw, Poland; 3 Department of Neurosurgery, Wroclaw Medical University, 50-367 Wroclaw, Poland; rafal.zaluski@umw.edu.pl; 4 Institute of Heart Diseases, Wroclaw Medical University, 50-556 Wroclaw, Poland; anna.gozdzik@umw.edu.pl; 5 Anesthesia and Intensive Care, San Martino Policlinico Hospital, IRCCS for Oncology and Neurosciences, 16132 Genoa, Italy; kiarobba@gmail.com; 6 Department of Surgical Sciences and Integrated Diagnostics (DISC), University of Genoa, 16145 Genoa, Italy

**Keywords:** cerebrospinal fluid, biomarkers, cardiac complications, subarachnoid haemorrhage, cerebral autoregulation, baroreflex

## Abstract

The pathophysiological mechanisms underlying severe cardiac dysfunction after aneurysmal subarachnoid haemorrhage (aSAH) remain poorly understood. In the present study, we focused on two categories of contributing factors describing the brain–heart relationship. The first group includes brain-specific cerebrospinal fluid (CSF) and serum biomarkers, as well as cardiac-specific biomarkers. The secondary category encompasses parameters associated with cerebral autoregulation and the autonomic nervous system. A group of 15 aSAH patients were included in the analysis. Severe cardiac complications were diagnosed in seven (47%) of patients. In the whole population, a significant correlation was observed between CSF S100 calcium-binding protein B (S100B) and brain natriuretic peptide (BNP) (r_S_ = 0.62; *p* = 0.040). Additionally, we identified a significant correlation between CSF neuron-specific enolase (NSE) with cardiac troponin I (r_S_ = 0.57; *p* = 0.025) and BNP (r_S_ = 0.66; *p* = 0.029), as well as between CSF tau protein and BNP (r_S_ = 0.78; *p* = 0.039). Patients experiencing severe cardiac complications exhibited notably higher levels of serum tau protein at day 1 (0.21 ± 0.23 [ng/mL]) compared to those without severe cardiac complications (0.03 ± 0.04 [ng/mL]); *p* = 0.009. Impaired cerebral autoregulation was noted in patients both with and without severe cardiac complications. Elevated serum NSE at day 1 was related to impaired cerebral autoregulation (r_S_ = 0.90; *p* = 0.037). On the first day, a substantial, reciprocal correlation between heart rate variability low-to-high frequency ratio (HRV LF/HF) and both GFAP (r_S_ = −0.83; *p* = 0.004) and S100B (r_S_ = −0.83; *p* = 0.004) was observed. Cardiac and brain-specific biomarkers hold the potential to assist clinicians in providing timely insights into cardiac complications, and therefore they contribute to the prognosis of outcomes.

## 1. Introduction

Aneurysmal subarachnoid haemorrhage (aSAH) is a severe subtype of stroke which may affect younger patients, and is marked by neurological and systemic complications that considerable influence morbidity and mortality rate. The mortality after aSAH is estimated at approximately 40%, with a 1.5-times elevated risk of post-survival fatality compared to the general population at the 12-month follow-up. Notably, this risk is attributed to concurrent cardiovascular and cerebrovascular complications [1,2].

Many survivors, including those who achieve a good functional outcome, contend with long-term neurological and psychosocial impairments [3,4]. The most crucial factors influencing functional outcomes are early brain injury (EBI), which emerges within the initial 72 h after aSAH, and delayed cerebral ischemia (DCI), representing a continuum of pathophysiological mechanisms that occurred between 4 and 14 days after ictus [5]. In addition to neurologic complications, patients with aSAH often have general and cardiopulmonary complications that can impact on outcomes [6,7,8]. Those cardiac events vary in clinical significance, ranging from subclinical electrocardiographic changes to cardiac troponin I (cTnI) leakage, severe heart failure with ventricular dysfunction, or cardiac arrest [9,10,11,12]. One of the most serious complications after aSAH is Takotsubo Syndrome (TTS). According to various studies, the incidence of TTS in aSAH ranges from 0.1 to 15% with a significant prevalence in postmenopausal women and patients with large ruptured aneurysms and low clinical grade [13,14,15]. The cardiac dysfunction, representing a manifestation of brain–heart axis disorders, has the potential to cause secondary brain damage. The pathophysiological mechanism underlying these alterations remains poorly understood, with potential clinical consequences including impaired cerebral perfusion pressure and cerebral hypoperfusion, the emergence of cerebral cardioembolic complications, oxygenation disorders, neurohormonal mechanisms, disruptions in the blood–brain barrier, systemic and cerebral inflammation, as well as activation of glial cells [16,17,18,19].

In recent years, there has been increased interest in markers associated with brain damage in the course of various neurological diseases, including aSAH. Their appearance in CSF or blood serum is attributed to brain damage or dysfunction, including neuronal and glial cells, increased permeability of the blood–brain barrier, or both. They are a valuable adjunct to radiological or clinical evaluation of the disease, providing insight into disease severity, lesion dynamics or risk stratification. In the present study, we focused on two distinct categories of factors, related to brain damage: the first being biomarkers, and the second, parameters associated with cerebral autoregulation and the autonomic nervous system (ANS). The first group includes brain-specific cerebrospinal fluid (CSF) and serum biomarkers: Glial Fibrillary Acidic Protein (GFAP) [20], tau proteins (TAU) [21], neuron-specific enolase (NSE) [22], and S100 calcium-binding protein B (S100B) [23], as well as cardiac-specific biomarkers [24]: creatine kinase-MB (CK-MB), and brain natriuretic peptide (BNP). Multiple research investigations have demonstrated a correlation between increased levels of proteins originating from both neuronal and glial cells and the occurrence of cellular brain damage in individuals diagnosed with aSAH. The secondary category encompasses parameters associated with cerebral autoregulation (pressure reactivity index, PRx) [25], and baroreflex sensitivity (BRS) [26], which serves as a surrogate of cardiovascular ANS regulations [27,28]. Both cerebral autoregulation and ANS hold the potential to assist clinicians in providing timely insights into patient clinical conditions, thereby aiding in the stratification of patient severity and the prognosis of outcomes.

The aim of our study was to compare brain-specific and cardiac-specific biomarkers along with autonomic dysfunction in a group of patients with severe cardiac complications after aSAH.

## 2. Materials and Methods

### 2.1. Patients

This retrospective single-centre study included patients who were treated for aSAH and were admitted to the Intensive Care Unit of Wroclaw University Clinical Hospital from January 2014 to December 2017. Written informed consent was obtained from all surviving patients, as approved by the local Bioethical Committee (approval KB-688/2014). Patients who did not survive were exempt from the requirement to obtain consent for the study, as approved by the local Bioethical Committee (approval KB-620/2020). The study was conducted in accordance with the Declaration of Helsinki.

Inclusion criteria comprised individuals aged 18 years or older, admitted to the hospital within 24 h of the manifestation of clinical symptoms, and undergoing aneurysm clipping or coiling within the initial 42 h post-admission. Criteria for enrolment in this study were the presence of clinically acute hydrocephalus requiring CSF drainage at the time of diagnosis, and the placement of an external ventricular drain (EVD) with subsequent collection of CSF samples between 1 to 3 days following aSAH. Exclusion criteria comprised cases of aSAH associated with mycotic aneurysm, arteriovenous malformation or trauma, along with patients with any history of neurological disease, tumours, complications of coiling or clipping, and patients admitted for diagnosis of cerebral death.

### 2.2. Clinical Evaluation

Patients with SAH were prospectively followed up throughout their hospitalization, with outcomes assessed both at the time of hospital discharge and after 12 months. Baseline characteristics, including demographics and previous medical histories, were documented for all patients. The patient’s clinical condition was evaluated using the Glasgow Coma Scale (GCS), the Acute Physiology and Chronic Health Evaluation II (Apache II) scale and the World Federation of Neurosurgical Societies (WFNS_ scale, graded from 1 (best) to 5 (worst) [29]. The severity of subarachnoid haemorrhage was categorized based on the modified Fisher scale (mFisher) (radiographic severity ranging from 0 to 4) [30] and Hijdra total score [31]. The Subarachnoid Haemorrhage Early Brain Edema Score (SEBES) [32] was employed to assess the extent of early cerebral oedema, with scores of 3–4 indicating high severity. Additionally, details such as the aneurysm’s location, presence of intraparenchymal haematoma, aneurysm treatment (clipping or coiling), as well any hospitalization-related complications (including vasospasm, and cardiac complications) were documented.

### 2.3. Blood and CSF Biomarkers

Levels of brain injury biomarkers were measured in venous blood and CSF samples on day 1 and in the following 48 h at 24 h intervals. Blood samples were collected using an intravenous catheter and, after centrifugation for 10 min at 2000× *g* at 4 °C, supernatants were stored at −80 °C. At the same time, CSF samples were collected through an EVD, centrifuged (10 min, 2000× *g* at 4 °C), and the supernatant was divided into portions and stored at −80 °C. An ELISA assay was used to measure the concentration of S100B (Cloud-Clone Corp., Katy, TX, USA), GFAP (Elabscience, Houston, TX, USA), TAU (R&D Systems, Minneapolis, MN, USA) and NSE (R&D Systems, Minneapolis, MN, USA). The concentrations of the biomarkers were analysed in duplicate with appropriate controls, according to the manufacturer’s instructions, using a microplate reader (ELx800 absorbance, BioTek, Winooski, VT, USA). In this analysis, we included levels of GFAP, TAU, NSE, and S100B on day 1, and maximum level from days 1–3. In addition, on the corresponding days, the values of parameters routinely measured in the hospital laboratory were recorded. Cardiac-specific biomarkers were measured upon admission to the hospital and on subsequent days of stay. Chemiluminescent microparticle immunoassay (CMIA) (Architecy STAY High Sensitivity Troponin I) was used to quantify cTnI in plasma. In this analysis, we included cTnI on day 1 and maximum level from days 1–3. Other laboratory results, such as white blood cells (WBC), high-sensitivity C-reactive protein (CRP), D-dimer, procalcitonin (PCT), blood lactate level (LAC), serum albumin, and blood glucose were measured on admission (within 24 h after the initial haemorrhage) and before treatment of the aneurysms. Biochemical parameters were divided into three groups: (1) brain-specific CSF and serum biomarkers: GFAP, TAU, NSE, and S100B; (2) cardiac-specific serum biomarkers: Creatine kinase-MB (CK-MB), cTnI, and brain natriuretic peptide (BNP); (3) non-specific serum biomarkers: white blood cells (WBC), high sensitivity C-reactive protein (CRP), procalcitonin (PCT), D-dimer, haematocrit (Ht), electrolytes (sodium (Na), potassium (K)), albumin, blood glucose and lactate levels (LAC).

### 2.4. Treatment and Signal Monitoring

All patients were managed within the intensive care unit in accordance with the institute’s standard protocol for aSAH patient care. This includes an early provision of aneurysm management, administration of nimodipine, and symptomatic treatments such as the maintenance of proper blood pressure, mechanical ventilatory support, administrating proper fluid infusion, utilizing analgesic and sedative drugs, and employing anti-oedema therapy when necessary to reduce intracranial pressure. The decision regarding the placement of an intracranial pressure sensor to monitor intracranial pressure (ICP) and cerebral perfusion pressure (CPP) was collaboratively determined by the neurosurgeons and neuro intensivists according to current guidelines [33]. This decision relied upon the radiological assessment, intraoperative conditions, and the patient’s clinical status. A control CT scan was conducted for all patients within 24 h following the coiling or clipping of the aneurysm. Additionally, further follow-up CT scans were performed during the hospital stay based on clinical indications. Neurological deficits or changes in the level of consciousness were assessed daily through physical examinations. Electrocardiography, transthoracic echocardiography, and plasma biochemical marker measurements were carried out at least once within the span of 1–4 days. Two-dimensional transthoracic echocardiography (Vivid 7 GE-Vingme) was conducted within 1–3 days. Routine monitoring for detecting CV included serial transcranial Doppler ultrasonography (TCD) measurements with a 2 MHz probe (Doppler BoxX, DWL Compumedics Germany GmbH, Singen, Germany). Clinical CV was defined as mean cerebral blood flow velocity (CBFV) in the middle cerebral artery exceeding 120 cm/s or a daily increase of CBFV of 20% when measured using TCD. DCI was defined as hypodensity on CT scan that had not been adjudicated as being due to surgical or endovascular intervention and the lesion was not due to EVD placement or intraparenchymal haematoma [34]. Neurogenic pulmonary oedema was defined as clinical manifestations of pulmonary involvement bilateral infiltrate in the chest X-ray and partial pressure oxygen/fractional inspired oxygen concentration (PaO_2_/FIO_2_) less than 200, as well no evidence of left atrial hypertension and absence of other common causes of acute respiratory distress [35].

Multimodal signal recording was performed using the Intensive Care Monitor (ICM+) computer system with a sampling frequency of 200 Hz. Signal monitoring was performed continuously during the patient’s stay in the ICU; however, in this analysis day 1 and day 3 were included. The arterial blood pressure (ABP) was measured invasively in the radial or femoral artery using a pressure transducer (Argon Standalone DTX Plus™, Argon Medical Devices Inc. Plano, TX, USA). ICP was measured invasively using an intraparenchymal sensor (Codman MicroSensor ICP Transducer, Codman & Shurtleff, Raynham, MA, USA) inserted into the frontal cortex. ABP was measured in the radial or femoral artery using standard monitoring kits (Baxter Healthcare, Cardiovascular Group, Irvine, CA, USA). Cerebral autoregulation was evaluated using a pressure reactivity index (PRx). PRx was calculated as the Pearson linear correlation coefficient between slow waves in the ABP and ICP signal [36]. First, the signals were averaged over 10 s intervals to isolate the slow changes, and then the correlation coefficient was assessed in 5 min moving average windows updated every 10 s. PRx > 0.3 indicates impaired cerebral autoregulation [37]. Two parameters were detrained as autonomic nervous system (ANS) metrics: baroreflex sensitivity (BRS), and heart rate variability (HRV). BRS was assessed in the time domain based on the sequential cross-correlation method proposed by Westerhof et al. [38] using built-in functions of ICM+ software (ver. 16). It was calculated as the slope of the regression line between 10 s segments of the systolic peak-to-peak interval and the corresponding systolic pressure time series derived from the ABP signal. HRV was assessed in the frequency method using the Lomb–Scargle periodogram and the ratio between low range (LF, 0.04–0.15 Hz) and high range (HF, 0.15–0.40 Hz) (HRV LF/HF) was calculated [39].

### 2.5. Endpoints and Definitions

The primary outcome of our study was an occurrence of severe cardiac complications within first three days. Cardiac complications were diagnosed based on elevated cardiac enzymes, changes in the electrocardiogram and cardiac dysfunction confirmed by transthoracic echocardiography. Based on the classifications of Sposato et al. [37], cardiac complications were divided into the following groups: (1) non-ischemic increased cTnI levels, (2) post-stroke acute myocardial infarction (AMI), (3) LV dysfunction, heart failure (HF) and post-stroke TTS; (4) ECG changes and arrhythmias. The patients were diagnosed with TTS according to the criteria of The International Takotsubo (Inter TAK) diagnostic criteria: the presence of a transient regional wall motion abnormality of the left ventricle that is thought to extend beyond a single epicardial coronary vascular distribution, the presence of new ST-segment elevation and/or T-wave inversion or modest elevation in cardiac biomarkers; the absence of significant obstructive coronary artery disease and the absence of myocarditis. The presence of coronary artery disease per se was not considered an exclusion criterion for the InterTAK Diagnostic Score [38].

The secondary outcome was assessed at discharge from the hospital using the modified Rankin Scale (mRS). A good outcome was defined as 0–2 on the mRS and poor as 3–6 on the mRS [40].

## 3. Results

### 3.1. Patients Characteristics

**Figure 1:**
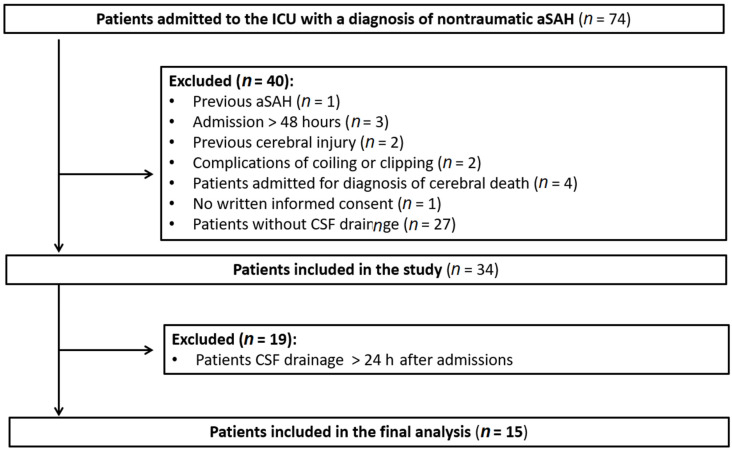
Flow chart. Abbreviations: ICU, intensive care unit; aSAH, aneurysmal subarachnoid haemorrhage; CSF, cerebrospinal fluid.

Between April 2014 and September 2017, 74 patients with non-traumatic aSAH were admitted to the Intensive Care Unit of Wroclaw University Hospital and were assessed for inclusion. Of these, 15 were included in this study (Figure 1). A poor outcome was observed in six (40%) of the patients. There were no significant differences in the baseline characteristics between those with a good and poor outcome (Table 1). The length of stay in the ICU and hospital was comparable in both groups. Univariate analysis revealed that a higher initial WFNS grade (*p* = 0.035) and Apache II scale score (*p* = 0.049) were significantly associated with a poor functional outcome. DCI occurred in four patients, all of whom experienced poor outcomes. A total of nine patients (60%) underwent aneurysm clipping due to their poor neurological status, indicated by the presence of intracerebral haematoma and signs of intracranial hypertension. In nine patients, ICP and CPP were monitored. Among these, three patients were managed conservatively after the placement of an EVD. The aneurysm closure procedure was postponed for some patients due to their poor neurological and clinical condition as well as their age. Unfortunately, due to extensive cerebral damage, these patients did not survive their ICU stay.

In terms of cardiac complications, thirteen (87%) patients exhibited new ECG changes within the first three days. Abnormal cTnI levels were observed in eight (53%) of the patients, and increased BNP levels were noted in seven (47%) of them. Echocardiography revealed abnormalities in thirteen (87%) patients, with TTS confirmed in six patients. Among these, four patients were discharged home. Two patients experienced in-hospital cardiac arrest as the cause of Pulseless Electrical Activity (PEA). Severe cardiac complications were diagnosed in seven (47%) of patients. Fluid therapy and circulatory support were administered based on hemodynamic measurements. Six patients with TTS and CV were treated with milrinone and low doses of norepinephrine, while one patient with TTS without CV was treated with milrinone only. In the remaining group with CV, only norepinephrine was used. All patients underwent a chest X-ray, which revealed changes: congestion in four (27%) patients and pulmonary oedema in five (33%) patients. Patient characteristics are presented in Table 1.

### 3.2. Relationship between Cardiac Complications and DCI

Within a subset of seven patients who experienced serious cardiac complications, CV was observed in six patients (6/7; 86%), whereas DCI was diagnosed in two subjects (2/7; 29%).

### 3.3. Biomarkers vs. Cardiac Complications

**Figure 2:**
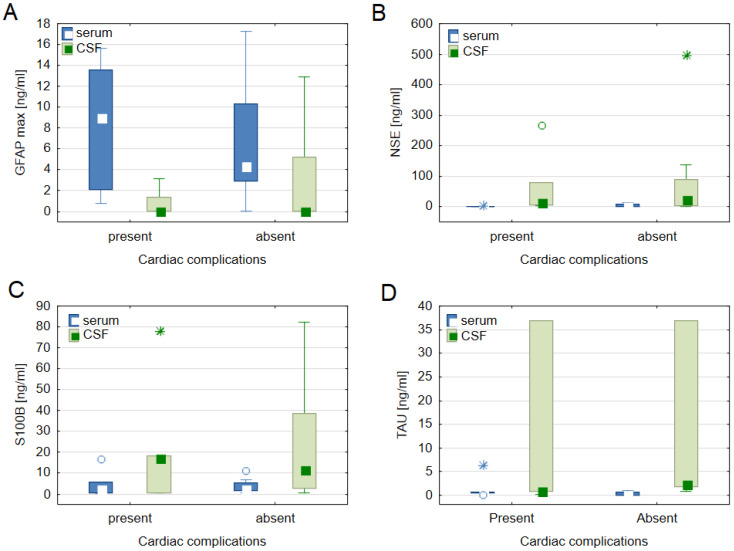
Median values with an interquartile range for the maximum concentration of the brain-specific biomarkers: Glial Fibrillary Acidic Protein (GFAP) (**A**), Neuron-Specific Enolase (NSE) (**B**), S100 Calcium-Binding Protein B (S100B) (**C**), and Tau Proteins (TAU) (**D**) in patients with and without severe cardiac complications after subarachnoid haemorrhage (aSAH). A circle represents an outlier, and an asterisk represents an extreme value.

Table 2 presents the average concentration of biomarkers related to cardiac disease, brain damage, and non-specific serum indicators for the entire patient cohort. In comparison to normal values (as detailed in Table 2), elevated levels of nearly all biomarkers were observed in patients following aSAH. Notably, patients with severe cardiac complications exhibited significantly higher levels of serum TAU on day 1 (0.21 ± 0.23 ng/mL) compared to those without severe cardiac complications (0.03 ± 0.04 ng/mL); *p* = 0.009. However, no significant differences were found in terms of cardiac biomarkers and non-specific biomarkers between patients with and without severe cardiac complications. Figure 2 presents a comparison of the maximal concentration of brain biomarkers and cardiac complications.

### 3.4. Cardiac Disease Biomarkers and Brain Damage Biomarkers

The correlations between cardiac biomarkers and brain-specific biomarkers are summarized in Table 3. We observed a significant correlation between CSF S100B on day 1 and BNP (r_S_ = 0.62; *p* = 0.040). Additionally, we found correlations between the maximum CSF NSE level and maximum cTnI (r_S_ = 0.57; *p* = 0.025), as well as BNP (r_S_ = 0.66; *p* = 0.029). Furthermore, there was a significant association between the maximum CSF TAU on day 1 and BNP (r_S_ = 0.78; *p* = 0.039). The scatter plots depicting the significant relationship are presented in Supplementary Figure S1.

### 3.5. ANS and Cerebral Autoregulation vs. Severe Cardiac Complications

**Figure 3:**
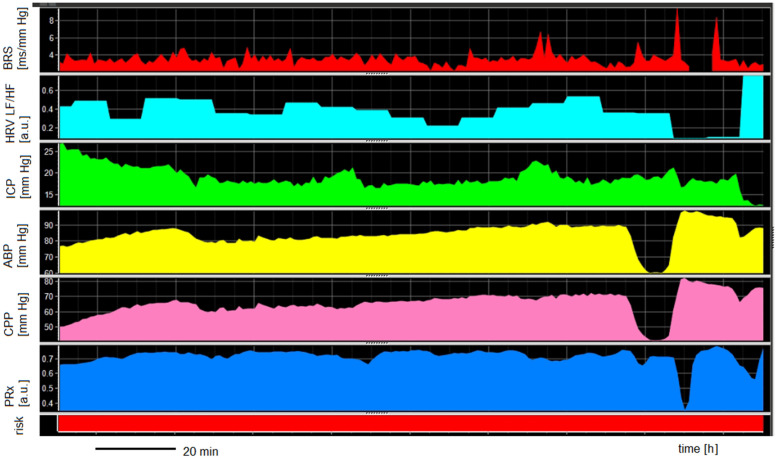
An example of multimodal monitoring in patients with cardiac complications (Takotsubo syndrome). Abbreviations: ABP, arterial blood pressure; ICP, intracranial pressure; CPP, cerebral perfusion pressure; PRx, pressure reactivity index; BRS, baroreflex sensitivity; HRV LF/HF, heart rate variability as the ratio between low-frequency range (LF, 0.04–0.15 Hz) and high-frequency range (HF, 0.15–0.40 Hz); risk, PRx > 0.3, which refers to impaired cerebral autoregulation.

An example of multimodal monitoring in aSAH patients with cardiac complications is presented in Figure 3. A comparison of ANS and cerebral autoregulation between patients with and without severe cardiac complications is presented in Table 4. Every patient exhibited impaired cerebral autoregulation (PRx > 0.3). This disturbance was observed both on day 1 (PRx: 0.7 ± 0.1) and day 3 (PRx: 0.6 ± 0.2). Although we noted higher BRS on days 1 and 3 in patients without severe cardiac complications compared to those with such complications, this disparity did not attain statistical significance (*p* = 0.248 and *p* = 0.533, respectively).

### 3.6. ANS and Cerebral Autoregulation vs. Biomarkers

The relationship among the ANS, cerebral regulation, and biomarkers is outlined in Table 5. On the first day, a substantial connection between HRV LF/HF and both GFAP (r_S_ = −0.83; *p* = 0.004) and S100B (r_S_ = −0.83; *p* = 0.004) was observed. Additionally, a significant correlation emerged between NSE on day 1 and PRx on day 1 (r_S_ = 0.90; *p* = 0.037). In terms of maximal concentrations, a significant association was found between the maximum NSE level and PRx on day 1 (r_S_ = 0.90; *p* = 0.037), as well as between maximum NSE and BRS on day 3 (r_S_ = 0.57; *p* = 0.042). Furthermore, the maximum CSF GFAP concentration exhibited a significant correlation with PRx on day 3 (r_S_ = 0.72; *p* = 0.027), while the maximum S100B concentration was linked to HRV LF/HF on day 1 (r_S_ = −0.66; *p* = 0.038). In relation to cardiac biomarkers, a significant association was observed between the highest BNP level and PRx (r_S_ = 0.90; *p* = 0.037). Scatter plots in Supplementary Figure S2 depict the correlation between the autonomic nervous system and biomarkers, while Supplementary Figure S3 illustrates the correlation between cerebral autoregulation and biomarkers.

## 4. Discussion

In our preliminary study, we utilized a combination of brain-specific and cardiac-specific biomarkers, along with physical parameters during the acute phase of brain injury, to predict the development of cardiac complications in patients with aSAH. This analysis focuses on the bidirectional nature of the brain–heart axis. While cardiac complications may not significantly impact treatment outcomes, they hold importance as they can potentially exacerbate initial pathological changes and provide a better understanding of ANS after aSAH. Therefore, exploring the pathophysiological mechanisms of cardiac complications following aSAH may help define the therapeutic window during the acute phase of aSAH.

The extent of brain damage and the development of DCI, which is regarded as a continuum of changes occurring during the acute phase following an event, are considered the primary causes of poor outcomes in patients with aSAH. Haemorrhage-related abnormalities, brain oedema, increased ICP, reduced cerebral blood flow, and impaired autoregulation are among the many affected areas. Additionally, brain and systemic inflammation, impairment of the neurovascular unit leading to increased blood–brain barrier permeability, microthrombosis of small vessels, and cortical depolarization are implicated. Autonomic dysfunction and inflammatory response not only manifest within the intracranial space but also affect other organs, including the cardiovascular system [41,42,43]. Cardiovascular complications symptomatic of disruption in the brain–heart axis are not the decisive factor for the final outcome but are independently associated with an elevated risk of DCI, cerebral infarction, and unfavourable outcome [7,11,44]. Furthermore, studies have demonstrated that these complications, particularly conditions such as TTS, increase the risk of death and subsequent cardiovascular events [45,46].

Cardiac dysfunction can arise from several mechanisms, including impaired regulation of the autonomic system, catecholamine release, activation of the hypothalamic–pituitary–adrenal axis, dysbiosis of the gut microbiome, inflammation, and immune responses. Additionally, the release of microvesicles by damaged astrocytes, neurons, microglia, and microRNA may contribute to these processes [19,47,48]. Clinically, the spectrum of changes referred to as Stroke–Heart Syndrome encompasses electrocardiographic alterations and arrhythmias, acute myocardial damage indicated by elevated cTnI, left ventricular dysfunction, heart failure, TTS, acute myocardial infarction, or sudden cardiac death [49,50]. In our study, ECG changes and elevated cTnI levels were observed in 87% and 53% of patients, respectively. These findings are consistent with prior research demonstrating their prevalence within this patient group [6,11,43].

In our study, we also investigated the correlation between levels of brain- and cardiac-damage biomarkers. Across the entire study group, the examined biomarkers of brain damage exhibited elevation, particularly in the CSF. In patients with poor outcomes, there was a notable elevation even in blood samples, suggesting that these markers reflect not only the extent of brain damage during the acute phase of the disease but also the degree of disruption to the blood–brain barrier [51,52,53]. Several studies have shown that elevated levels of proteins from neuronal and glial cells damage are associated with cellular brain damage in patients with aSAH [21], TBI, ischemic strokes [54,55,56,57] or cardiac arrest [58,59]. When studying the relationship between biomarkers of cerebral and cardiac damage, we found the strongest relationship between the levels of NSE, TAU protein and S100B in CSF.

NSE protein is a highly specific marker for neurons; depending on the region, it accounts for 0.4% to 2.2% of total soluble brain protein. It is also a specific marker of peripheral neuroendocrine tissue. The presence of NSE has been demonstrated in granule cells, Purkinje cells, projection neurons and both sensory and autonomic neurons. In addition, its presence was demonstrated in pinealocytes, pituitary glandular and peptide-secreting cells, thyroid parafollicular cells, adrenal medullary chromaffin cells, cells of the islets of Langerhans, Merkel’s cells of the skin, neuroendocrine cells of the lung, and erythrocytes. Studies have shown that increased levels of NSE in cerebrospinal fluid or serum may have value in diagnosing the degree of brain damage [60,61,62,63]. NSE has been shown to provide quantitative measurements of brain damage and improve diagnosis and outcome assessment in seizures, traumatic brain injury, and ischemic strokes. In a study including patients with aneurysmal subarachnoid haemorrhage, NSE levels in both cerebrospinal fluid and serum were found to have predictive value for mortality and long-term outcomes [64,65,66]. Importantly, the biological half-life of NSE in body fluids is about 24 h, so the persistence of high levels of NSE or even an increase in subsequent days may indicate ongoing pathophysiological processes leading to further damage to neuronal cells [67]. A limitation of our study is the lack of assessment of NSE at a distant time after ictus, but the specific time frame of our study could provide a therapeutic window for inhibiting these changes.

Another protein that showed a correlation with cardiac biomarkers was TAU protein. TAU is an intracellular microtubule-associated protein that is highly concentrated in axons and is involved in the formation of axonal microtubule bundles and is also involved in axoplasmic transport [67]. Changes in TAU are apparently associated with axonal disruption, and damage to its integrity can lead to the release of TAU into the extracellular space. The TAU protein cleaves and diffuses into the CSF and, depending on the integrity of the blood–brain barrier, into the blood in variable amounts [68]. Previous studies have shown that elevated CSF-TAU levels are associated with cellular brain damage in patients with SAH [21,69]. In a study by Helbok at al. using brain microdialysis, it was shown that TAU protein levels in CSF, after an initial 24 h fluctuation, remained elevated throughout the monitoring period (9–14 days) and correlated with poor long-term outcomes [21]. Another study highlighted the role of neuroinflammation with TAU protein levels, metabolic disorders, or the development of DCI [69]. Studying the dynamics of changes in TAU, the authors found that TAU values remained high in patients with delayed cerebral ischemia and poor 12-month outcomes [70]. Considering the half-life of the tau protein (115 h), as the cause of persistently elevated TAU levels, the authors suggest ongoing axonal damage or impaired axonal integrity secondary to complex EBI mechanisms. Similarly, the work of Zanier et al. showed an association between TAU protein levels, cerebral vasospasm, and long-term outcome [71]. The association of TAU with cardiac biomarkers in our study reflects both the extent of brain damage and its impact on the brain–heart axis.

We chose S100B as a biomarker of both brain and heart damage. S100B, a Ca^2+^-binding protein, functions as a regulator of intracellular activities and as an extracellular signal. S100B protein in the highest concentrations is present in astroglial cells as well as adipocytes, striated muscle, intestinal glial cells, adipocytes, chondrocytes, melanocytes, arterial smooth muscle cells and bronchial epithelium and cardiac muscle. S100B expression can be induced in cardiomyocytes and arterial endothelium in response to norepinephrine. Our results show that CSF S100B change correlated with cTnI, BNP and severity of cardiac complications and mortality. In addition, in three patients with good outcomes, with TTS, serum S100B levels were higher than those in CSF, which may indicate an extracerebral source of S100B, possibly the heart [72,73,74]. This also supports the idea that although left ventricular dysfunction can increase the risk of complications, such as cerebral infarction, due to hypotension and vasospasm, when recognized and treated early, it does not have a major impact on outcomes [44]. S100B levels are detectable in high concentrations in both cerebrospinal fluid and blood in patients with acute cerebral injury in the course of cardiovascular conditions, or trauma. They have predictive value for predicting outcomes in various clinical conditions, measured against radiological criteria, clinical status or functional outcomes. The role of glial cells in the development of cardiovascular disease has also been raised, pointing to disorders of the brain–heart axis, where astrocytes and peripheral glial cells are key modulators, affecting sympathetic nervous system activity (central and peripheral [75]).

The mechanism of ANS disorders following aSAH remains unknown, including molecular mechanisms, which are still unexplored [26,27,28]. Previous studies have emphasized the role of the cholinergic system in central nervous system diseases, where acetylcholine (ACh) serves as a crucial mediator in the communication between the brain and the immune system. The regulatory function of ACh in response to brain injury involves immune cells within the brain, influencing the function of the blood–brain barrier (BBB) and the extent of systemic immune response. An imbalance between pro- and anti-inflammatory processes can not only modify the immune activity of brain microglia, astrocytes, or perivascular macrophages, but affect the increase in BBB permeability, leading to the influx of peripheral immune cells and subsequent neuronal damage. The systemic immune response is dependent on the regulation of the parasympathetic and sympathetic nervous system, as well as the hypothalamus–pituitary–adrenal glands axis (PHA) [76].

Impaired baroreflex sensitivity serves as a surrogate measure indicating the downregulation of the parasympathetic (PNS) system. A study by Winek at al. highlights the role of regulators of cholinergic signalling regulators as potential biomarkers of increased risk of infectious complications [77]. In our study, we observed a tendency toward higher BRS on days 1 and 3 in patients without cardiac complications compared to those with such complications. This finding aligns with previous research on ANS changes in aSAH. It has been shown that lower BRS is associated with more extensive haemorrhage, worse outcomes, and an increased risk of death [28]. Moreover, aSAH induces a decrease in sympathetic and total autonomic cardiovascular modulation. In our analysis, we also identified a significant, reciprocal correlation between HRV LF/HF and both GFAP and S100B, indicating that elevated levels of these brain-specific biomarkers were associated with a diminished HRV LF/HF ratio. The LF/HF ratio is regarded as an indicator of the directional change in sympathovagal activity. The link between LF/HF ratio and cardiac or neurologic complications after aSAH was shown in previous studies [78,79,80].

In our study, we found that cerebral autoregulation was impaired in the entire cohort of aSAH patients. PRx was correlated with NSE and GFAP; therefore, impaired cerebral autoregulation (indicated by a higher PRx) was associated with increased levels of NSE and GFAP. This observation is consistent with our previous research, where we found a positive relationship between NSE and cerebral autoregulation [81]. Previous studies have also indicated that cerebral autoregulation is related to inflammation-related blood biomarkers [82].

Previous studies have demonstrated that cognitive complaints are correlated with cognitive deficits, anxiety, and depression, impacting activities of daily living [83,84,85]. The study by Nobels-Janssen et al. recommends comprehensive testing for both subjective cognitive complaints and objective cognitive functioning [86]. The authors emphasize that patient-reported cognitive complaints may indicate cognitive dysfunction, suggesting screening (using the ‘Symptom Screening Questionnaire for Aneurysmal Subarachnoid Hemorrhage’) and subsequent neuropsychological testing in aSAH patients. Another concern is the potential impact of anticholinergic drugs, which encompass a wide range of medications, on the development of cognitive impairment. This is particularly relevant in patients with stroke and multimorbidity. Although aSAH primarily affects younger patients compared to the ischemic stroke population, chronic use of these drugs deserves attention. In our study, a significant number of patients reported decreased quality of life, emotional disturbances, or apathy. Notably, only one patient had been using an anticholinergic antidepressant drug for more than three months. As the main aim of this study was to analyse the relationship between biomarkers and cardiac dysfunctions, we showed only mRankin scores and did not present the results of the SF-36 questionnaire. However, in our next study, we plan to analyse cognitive dysfunction and biomarker dynamics in subarachnoid haemorrhage patients.

It is worth noting that modern biological sciences are constantly evolving, offering new opportunities to study not only individual biomarkers but also to track entire regulatory pathways systemically and investigate the long-term consequences of their mutual dependencies. Increasing efforts are directed toward integrating genomics research with other omics technologies, including transcriptomics and proteomics, which enable the analysis of post-translational modifications and the characterization of specific biological or pathological processes, including those in the CNS. An example of this can be seen in the search for and tracking the role of micro-RNA molecules in the regulation of oxidative stress, as markers of blood–brain barrier damage, and as mediators of the brain’s inflammatory response [87].

There are some limitations to the present study. First, the number of subjects in our study was limited to the original EVD setting, resulting in a relatively small sample size. Second, the study is single-centre, and although data were collected prospectively, we performed the final analysis retrospectively. Another limitation is the absence of coronary angiography in patients with TTS; in practice, we relied on daily echocardiography and haemodynamic studies. Another limitation is the lack of measurement of biomarkers in the late phase of aSAH and the lack of evaluation of cardiovascular complications at 12 months. A limitation of our study is also the absence of a healthy control group for comparative analysis. Since our research is based on data from an aSAH-patient population, we were unable to include a group of healthy individuals for direct comparison. This limits conclusions about the observed biomarker changes in relation to the baseline healthy state. Nevertheless, our study provides valuable insights into the biomarker dynamics within the studied patient cohort, offering a foundation for future research that may include healthy controls.

## 5. Conclusions

Cardiac and brain-specific biomarkers may provide additional information about the pathogenesis of cardiac complications after aSAH, and therefore they contribute to the prognosis of outcomes. Further studies are needed to investigate the impact of cardiac and brain-specific biomarkers on the autonomic nervous system and cerebral autoregulation.

## Figures and Tables

**Table 1:** Baseline characteristics of the whole cohort of patients with aSAH versus outcome in modified Rankin scale (mRS) after 12 months. Data are presented as median ± interquartile range or number of subjects (%).

Characteristic	Total N = 15	mRS 0–2 *n* = 6	mRS 3–6 *n* = 9	*p*-Value
Age	67 ± 28	60 ± 18	74 ± 22	0.617
Female	8 (53%)	4 (67%)	3 (33%)	0.230
BMI	27.8 ± 9.3	30.2 ±12.6	27.8 ± 3.0	0.643
GCS	12 ± 10	14 ± 1	6 ± 8	0.071
MAP [mm Hg]	65 ± 33	58 ± 32	66 ± 23	0.982
HR [bpm]	80 ± 11	58 ± 32	65 ± 23	0.857
Temp [°C]	37.0 ± 1.1	37.0 ± 0.4	37.6 ± 0.9	0.642
Reactive pupils	15 (100%)	6 (100%)	9 (100%)	**-----**
Comorbidities				
Hypertension	8 (53%)	3 (50%)	5 (55%)	0.751
Hyperlipidaemia	7 (47%)	2 (33%)	5 (55%)	0.377
Current smoker	10 (67%)	4 (67%)	6 (67%)	0.713
Alcoholism	1 (7%)	0	1 (11%)	0.600
Diabetes mellitus	0	0	0	**-----**
Coronary heart disease	3 (20%)	0	3 (33%)	0.184
Aneurysm				
ICA	3 (20%)	2 (33%)	1 (11%)	0.565
MCA	2 (13%)	1 (17%)	1 (11%)	
ACA	1 (7%)	0	1 (11%)	
ACoA	6 (40%)	3 (50%)	3 (33%)	
Posterior	3 (20%)	0	3 (33%)	
Clinical assessment				
mFisher	4	4 ± 2	4	0.867
Apache II	18 ± 14	12 ± 6	20 ± 6	**0.049**
Hijdra	27 ± 17	22 ± 16	27 ± 11	0.529
SEBES	2 ± 3	2 ± 2	1 ± 3	0.857
SEBES: Grade 0–2	11 (73%)	5 (83%)	6 (67%)	0.461
SEBES: Grades 3–4	4 (27%)	1 (17%)	3 (33%)	
WFNS	4 ± 3	2 ± 1	5 ± 1	0.143
WFNS: Grade I-III	7 (47%)	5 (83%)	2 (22%)	**0.035**
WFNS: Grade IV-V	8 (53%)	1 (17%)	7 (78%)	
ICH	5 (33%)	1 (17%)	4 (44%)	0.293
IVH	15 (100%)	6 (100%)	9 (100%)	**-----**
Aneurysm treatment				
Clipping	9 (60%)	6 (100%)	3 (33%)	**0.016**
Coiling	3 (20%)	0	3 (33%)	0.184
Conservative treatment	3 (20%)	0	3 (33%)	0.184
Outcome				
ICU stay [days]	17 ± 14	13 ± 11	18 ± 15	0.170
Hospital stay [days]	18 ± 15	19 ± 3	18 ± 49	0.133
Dead	6 (40%)	0	6 (67%)	**0.016**
Neurologic complications				
Pathological changes in CT after surgery	12 (80%)	4 (67%)	8 (89%)	0.340
CV	10 (67%)	5 (83%)	5 (55%)	0.293
DCI	4 (27%)	0	4 (44%)	0.092
Cardiac complications				
non-ischemic increased troponin levels	8 (53%)	3 (50%)	5 (56%)	0.622
post-stroke AMI	0	0	0	------
LV dysfunction, HF and post-stroke TTS	7 (47%)	3 (50%)	4 (44%)	0.622
ECG changes and arrhythmias	13 (87%)	6 (100%)	7 (78%)	0.342
Pulmonary complications				
Pulmonary oedema	5 (33%)	2 (33%)	3 (33%)	0.706
Pulmonary congestion	4 (27%)	1 (17%)	3 (33%)	0.461

Abbreviations: ACA, anterior cerebral artery; ACoA, anterior communicating artery; AMI, acute myocardial infarction; BMI, body mass index; CT, computed tomography; CV, cerebral vasospasm; DCI, delayed cerebral ischemia; GCS, Glasgow Coma Scale; HF, heart failure; HR, heart rate; ICA, internal carotid artery; ICH, Intracerebral Haemorrhage; ICU, intensive care unit; IVH, Intraventricular Haemorrhage; LV, left ventricular; MAP, mean arterial pressure; MCA, middle cerebral artery; mFisher, modified Fisher scale; SEBES, The Subarachnoid Haemorrhage Early Brain Edema Score; TTS, Takotsubo Syndrome; WFNS grade, World Federation of Neurosurgical Societies grade; *p*-value was obtained using U Mann–Whitney test; significant differences are marked in bold.

**Table 2:** Biomarkers characteristics of the whole cohort of patients with aSAH versus severe cardiac complications. Data are presented as median ± interquartile range. The normal range is presented in brackets. If the biomarker was not evaluated in all patients, the exact number of subjects is marked with subscript and presented below the table.

Characteristic	Total N = 15	Severe Cardiac Complications *n* = 7	Without Severe Cardiac Complications *n* = 8	*p*-Value
Brain biomarkers				
serum GFAP day 1 [ng/mL]	4.38 ± 8.77	4.94 ± 9.54	2.83 ± 7.39	0.396
serum GFAP max [ng/mL]	4.50 ± 9.27	9.05 ± 11.47	4.41 ± 7.41	0.778
CSF GFAP day 1 [ng/mL]	0.03 ± 3.12	0.03 ± 3.12	0.07 ± 5.61	0.231
CSF GFAP max [ng/mL]	0.05 ± 3.12	0.05 ± 1.34	0.07 ± 5.16	0.612
serum S100B day 1 [ng/mL]	0.93 ± 3.21	0.93 ± 0.79	2.03 ± 3.49	0.535
serum S100B max [ng/mL]	2.30 ± 4.76	2.30 ± 5.00	2.31 ± 3.74	0.778
CSF S100B day 1 [ng/mL]	17.76 ± 23.77	17.76 ± 21.37	12.89 ± 35.58	0.612
CSF S100B max [ng/mL]	13.41 ± 24.91	17.76 ± 17.72	11.66 ± 36.16	0.778
serum NSE day 1 [ng/mL]	1.40 ± 5.00	1.40 ± 0.39	1.99 ± 7.78	0.280
serum NSE max [ng/mL]	1.81 ± 4.67	1.40 ± 0.67	3.67 ± 7.79	0.054
CSF NSE day 1 [ng/mL]	16.49 ± 68.70	7.60 ± 19.46	40.47 ± 100.51	0.396
CSF NSE max [ng/mL]	16.49 ± 76.57	16.49 ± 76.17	23.15 ± 85.63	0.866
^a^ serum TAU day 1 [ng/mL]	0.07 ± 0.19	0.21 ± 0.23	0.03 ± 0.04	**0.009**
^a^ serum TAU day max [ng/mL]	0.44 ± 0.60	0.45 ± 0.21	0.06 ± 0.60	0.177
^a^ CSF TAU day 1 [ng/mL]	2.05 ± 36.05	1.00 ± 36.05	2.33 ± 35.07	0.662
^a^ CSF TAU max [ng/mL]	2.05 ± 36.05	1.00 ± 36.05	2.33 ± 35.07	0.662
Cardiac biomarkers				
^b^ BNP [pg/mL] (0–100)	155 ± 119	155 ± 111	138 ± 116	0.792
cTnI day 1 [ng/mL] (0–0.028)	0.03 ± 0.66	0.04 ± 0.03	0.03 ± 1.55	0.955
cTnI max [ng/mL]	0.06 ± 1.97	0.06 ± 1.98	0.34 ± 2.08	0.955
CK-MB [U/L] (0–25)	28 ± 22	27 ± 25	32 ± 16	0.396
Non-specific biomarkers				
CRP [mg/L] (0–5)	5.20 ± 3.99	5.20 ± 6.98	4.35 ± 3.69	0.694
D-dimer [ug/mL] (0–0.5)	1.72 ± 3.16	2.35 ± 2.60	1.20 ± 4.06	0.120
WBC [G/L] (4–10 10^3^/ul)	14.83 ± 9.17	13.52 ± 12.67	15.01 ± 6.81	0.994
PCT [ng/mL] (0–0.05)	0.11 ± 0.28	0.11 ± 0.27	0.13 ± 0.28	0.778
LAC [mmol/L] (0.5–1.6)	1.75 ± 1.20	1.75 ± 0.70	1.58 ± 1.64	0.866
K [mmol/L] (3.5–5.0)	3.91 ± 0.70	4.10 ± 0.81	3.77 ± 0.42	0.463
Na [mmol/L] (136–146)	139 ± 3	139 ± 3	139 ± 3	0.694
Ht [L] (40–54)	43 ± 5	43 ± 5	43 ± 4	0.788
Glucose [mg/dL] (74–106)	186 ± 62	154 ± 50	202 ± 40	0.120
Albumin [G/L] (35–52)	30 ± 7	30 ± 12	30 ± 5	0.866

Abbreviations: GFAP, glial fibrillary acidic protein; CSF, cerebrospinal fluid; CK-MB, Creatine Kinase MB; BNP, B-type Natriuretic Peptide; cTnI, cardiac troponine type I; CRP, C-reactive protein; S100B, S100 calcium-binding protein B; Ht, haematocrit; K, potassium; Na, sodium; NSE, neuron specific enolase; WBC, white blood cell; LAC, lactate; PCT, procalcitonin; *p*-value was obtained using U Mann–Whitney test; significant differences are marked in bold; ^a^, TAU was evaluated in 11 patients; ^b^, BNP was evaluated in 11 patients.

**Table 3:** Spearman correlation between cardiac-specific biomarkers and brain-specific biomarkers in total group (N = 15) of patients with subarachnoid haemorrhage (aSAH). Significant correlations are marked with asterisk.

	Cardiac Biomarkers			
Brain-Specific Biomarkers	cTnI Day 1	cTnI Max	BNP ^a^	CK-MB
serum GFAP day 1	−0.14	−0.23	0.04	−0.04
serum GFAP max	−0.06	−0.15	−0.04	0.06
CSF GFAP day 1	0.05	0.10	0.31	0.16
CSF GFAP max	0.15	0.26	0.48	0.21
serum S100B day 1	0.01	0.02	0.22	0.04
serum S100B max	−0.04	0.07	0.55	−0.08
CSF S100B day 1	−0.21	−0.06	0.62 *	−0.29
CSF S100B max	−0.13	−0.02	0.62 *	−0.22
serum NSE day 1	−0.43	−0.38	−0.02	−0.26
serum NSE max	−0.43	−0.38	−0.02	−0.26
CSF NSE day 1	0.13	0.24	0.63 *	0.15
CSF NSE max	0.47	0.57 *	0.66 *	0.16
^b^ serum TAU day 1	0.25	0.18	−0.11	0.04
^b^ serum TAU max	0.33	0.29	0.53	−0.11
^b^ CSF TAU day 1	0.12	0.20	0.78 *	−0.16
^b^ CSF TAU max	0.12	0.20	0.78 *	−0.17

Abbreviations: GFAP, glial fibrillary acidic protein; CSF, cerebrospinal fluid; S100B, S100 calcium-binding protein B; NSE, neuron-specific enolase; CK-MB, Creatine Kinase MB; BNP, B-type Natriuretic Peptide; cTnI, cardiac troponie type I; max, maximal concentration from days 1–3; * *p* < 0.05; ^a^, BNP was evaluated in 11 patients; ^b^, TAU was evaluated in 11 patients.

**Table 4:** Cerebral autoregulation, cerebral haemodynamics and autonomic nervous system (ANS) parameters in the whole cohort of patients with aSAH versus cardiac complications. Data are presented as median ± interquartile range.

Parameter	Total Group * (N = 13)	Cardiac Complications (*n* = 7)	Without Cardiac Complications (*n* = 8)	*p*-Value
ABP day 1 [mm Hg]	83.1 ± 15.6	82.7 ± 15.59	90.4 ± 13.1	0.665
ABP day 3 [mm Hg]	87.9 ± 7.3	91.6 ± 13.8	86.3 ± 10.6	0.366
BRS day 1 [ms/mm Hg]	8.1 ± 6.0	6.2 ± 3.8	9.5 ± 4.5	0.248
BRS day 3 [ms/mm Hg]	8.6 ± 6.2	5.2 ± 9.4	9.1 ± 12.2	0.533
HRV LF/HF day 1 [a.u.]	0.9 ± 0.7	0.9 ± 0.3	0.9 ± 0.5	0.841
HRV LF/HF day 3 [a.u.]	1.0 ± 0.4	0.8 ± 0.4	1.1 ± 0.5	0.628
^a^ ICP day 1 [mm Hg]	7.7 ± 8.0	5.6 ± 2.7	8.9± 5.5	0.114
^a^ ICP day 3 [mm Hg]	11.2 ± 9.1	15.9 ± 10.3	7.8 ± 9.4	0.190
^a^ CPP day 1 [mm Hg]	70.7 ± 8.3	70.2 ± 30.5	70.7 ± 32.3	0.995
^a^ CPP day 3 [mm Hg]	78 ± 8.6	84.8 ± 18.5	77.6 ± 2.6	0.730
^a^ PRx day 1 [a.u.]	0.6 ± 0.1	0.7 ± 0.1	0.7 ± 0.1	0.200
^a^ PRx day 3 [a.u.]	0.7 ± 0.2	0.6 ± 0.2	0.8 ± 0.1	0.730

Abbreviations: ABP, arterial blood pressure; ICP, intracranial pressure; CPP, cerebral perfusion pressure; PRx, pressure reactivity index; BRS, baroreflex sensitivity; HRV LF/HF, heart rate variability as the ratio between low-frequency range (LF, 0.04–0.15 Hz) and high-frequency range (HF, 0.15–0.40 Hz); *, multimodal continuous recordings were available in *n* = 13 patients; ^a^; ICP, CPP, PRx were monitored in 9 patients (4 with and 5 without cardiac complications).

**Table 5:** Spearman correlation between cardiac-specific biomarkers, brain-specific biomarkers, and autonomic nervous system (ANS) as well cerebral autoregulation parameters in the total group (N = 13) of patients with subarachnoid haemorrhage (aSAH). Significant correlations are marked with an asterisk.

	ANS and Cerebral Autoregulation Parameters					
	BRS Day 1	HRV LF/HF Day 1	PRx ^a^ Day 1	BRS Day 3	HRV LF/HF Day 3	PRx ^a^ Day 3
Brain-specific biomarkers						
serum GFAP day 1	−0.18	−0.83 **	−0.60	0.16	−0.49	−0.56
serum GFAP max	−0.02	−0.57	−0.50	0.41	−0.34	−0.63
CSF GFAP day 1	−0.01	−0.59	0.35	0.04	−0.02	0.49
CSF GFAP max	−0.11	−0.51	−0.11	−0.25	−0.05	0.72 *
serum S100B day 1	−0.03	−0.78 **	0.01	0.43	−0.38	0.54
serum S100B max	−0.19	−0.66 *	0.10	0.25	−0.33	0.65
CSF S100B day 1	0.14	−0.29	0.56	0.34	−0.03	0.53
CSF S100B max	0.03	−0.03	−0.21	0.56	0.18	0.01
serum NSE day 1	0.30	0.33	0.90 *	0.59 *	0.32	0.07
serum NSE max	0.38	−0.15	0.90 *	0.57 *	0.05	0.42
CSF NSE day 1	0.45	−0.26	0.80	0.19	0.03	0.58
CSF NSE max	0.26	−0.25	0.05	0.04	0.04	0.03
^b^ serum TAU day 1	−0.47	−0.28	−0.56	−0.30	−0.27	0.03
^b^ serum TAU max	0.07	0.05	−0.60	−0.01	−0.05	−0.10
^b^ CSF TAU day 1	0.09	−0.13	0.50	0.44	−0.29	0.65
^b^ CSF TAU max	0.09	−0.13	0.50	0.44	−0.29	0.65
Cardiac-specific biomarkers						
cTnI day 1	0.28	0.01	0.15	−0.15	0.25	0.11
cTnI max	0.21	0.08	0.10	−0.31	0.13	0.08
^c^ BNP	0.57	−0.14	0.01	0.10	−0.03	0.90 *
CK-MB	−0.07	−0.29	0.30	−0.23	−0.35	−0.05

Abbreviations: GFAP, glial fibrillary acidic protein; CSF, cerebrospinal fluid; S100B, S100 calcium-binding protein B; NSE, neuron-specific enolase; cTnI, cardiac troponine type I; CK-MB, Creatine Kinase MB; BRS, baroreflex sensitivity; BNP, B-type Natriuretic Peptide; HRV LF/HF, heart rate variability as the ratio between low-frequency range (LF, 0.04–0.15 Hz) and high-frequency range (HF, 0.15–0.40 Hz); PRx, pressure reactivity index; max, maximal concentration from days 1–3; * *p* < 0.05; ** *p* < 0.01; ^a^, PRx was evaluated in 11 patients; ^b^, TAU was evaluated in 11 patients; ^c^, BNP was evaluated in 11 patients.

## Data Availability

The data presented in this study are available on request from the corresponding author. The data are not publicly available due to privacy restrictions.

## Supplementary Materials

The following supporting information can be downloaded at: https://www.mdpi.com/article/10.3390/biomedicines11102835/s1. Figure S1: Scatter plot presents correlation between: (A) B-type Natriuretic Peptide (BNP) and cerebrospinal fluid (CSF) S100 Calcium-Binding Protein B (S100B ) on day 1, (B) BNP and maximal concentration from days 1-3 (max) of CSF S100B, (C) BNP and CSF neuron-specific enolase (NSE) on day 1, (D) BNP and CSF NSE max, (E) cardiac troponine type I (cTnI) max and CSF NSE max, F) BNP and CSF TAU on day 1, (G) BNP and CSF TAU max. BNP was evaluated in 11 patients; TAU was evaluated in 11 patients; other biomarkers were evaluated in 15 patients. CSF refers to biomarker levels measured in cerebrospinal fluid; if not indicated biomarker levels were measured in serum. Figure S2. The scatter plots present a correlation between the autonomic nervous system (baroreflex sensitivity, BRS; heart-rate variability as the ratio between low-frequency range (LF, 0.04-0.15 Hz) and high-frequency range (HF, 0.15-0.40 Hz), HRV LF/HF) and biomarkers: glial fibrillary acidic protein (GFAP); neuron-specific enolase (NSE); S100 calcium-binding protein (S100B); if not indicated otherwise biomarker levels were measured in serum. Figure S3. The scatter plots present a correlation between the cerebral autoregulation (pressure reactivity index, PRx) and biomarkers: glial fibrillary acidic protein (GFAP); neuron-specific enolase (NSE); B-type Natriuretic Peptide (BNP); CSF refers to biomarker levels measured in cerebrospinal fluid; if not indicated biomarker levels were measured in serum. PRx were monitored in 9 patients. BNP was evaluated in 11 patients.

## References

[1] Huhtakangas J., Lehto H., Seppä K., Kivisaari R., Niemelä M., Hernesniemi J., Lehecka M. (2015). Long-Term Excess Mortality after Aneurysmal Subarachnoid Hemorrhage: Patients with Multiple Aneurysms at Risk. Stroke.

[2] Rinkel G.J.E., Algra A. (2011). Long-Term Outcomes of Patients with Aneurysmal Subarachnoid Haemorrhage. Lancet Neurol..

[3] Greebe P., Rinkel G.J.E., Hop J.W., Visser-Meily J.M.A., Algra A. (2010). Functional Outcome and Quality of Life 5 and 12.5 Years after Aneurysmal Subarachnoid Haemorrhage. J. Neurol..

[4] Dey S., Kumar J.K., Shukla D., Bhat D. (2018). Neurological, Neuropsychological, and Functional Outcome after Good Grade Aneurysmal Subarachnoid Hemorrhage. Neurol. India.

[5] Fujii M., Yan J., Rolland W.B., Soejima Y., Caner B., Zhang J.H. (2013). Early Brain Injury, an Evolving Frontier in Subarachnoid Hemorrhage Research. Transl. Stroke Res..

[6] Hravnak M., Frangiskakis J.M., Crago E.A., Chang Y., Tanabe M., John G.I., Horowitz M.B. (2009). Elevated Cardiac Troponin I and Relationship to Persistence of Electrocardiographic and Echocardiographic Abnormalities After Aneurysmal Subarachnoid Hemorrhage. Stroke.

[7] Van Der Bilt I.A.C., Hasan D., Vandertop W.P., Wilde A.A.M., Algra A., Visser F.C., Rinkel G.J.E. (2009). Impact of Cardiac Complications on Outcome after Aneurysmal Subarachnoid Hemorrhage: A Meta-Analysis. Neurology.

[8] Crago E.A., Kerr M.E., Kong Y., Baldisseri M., Horowitz M., Yonas H., Kassam A. (2004). The Impact of Cardiac Complications on Outcome in the SAH Population. Acta Neurol. Scand..

[9] Elsharkawy H., Abd-Elsayed A., El-Hadi S., Provencio J., Tetzlaff J. (2016). Fluctuating Electrocardiographic Changes Predict Poor Outcomes After Acute Subarachnoid Hemorrhage. Ochsner J..

[10] Burch G.E., Meyers R., Abildskov J.A. (1954). A New Electrocardiographic Pattern Observed in Cerebrovascular Accidents. Circulation.

[11] Naidech A.M., Kreiter K.T., Janjua N., Ostapkovich N.D., Parra A., Commichau C., Fitzsimmons B.F.M., Connolly E.S., Mayer S.A. (2005). Cardiac Troponin Elevation, Cardiovascular Morbidity, and Outcome after Subarachnoid Hemorrhage. Circulation.

[12] Jeon I.-C., Chang C.-H., Choi B.-Y., Kim M.-S., Kim S.-W., Kim S.-H. (2009). Cardiac Troponin I Elevation in Patients with Aneurysmal Subarachnoid Hemorrhage. J. Korean Neurosurg. Soc..

[13] Talahma M., Alkhachroum A.M., Alyahya M., Manjila S., Xiong W. (2016). Takotsubo Cardiomyopathy in Aneurysmal Subarachnoid Hemorrhage: Institutional Experience and Literature Review. Clin. Neurol. Neurosurg..

[14] Malik A.N., Gross B.A., Rosalind Lai P.M., Moses Z.B., Du R. (2015). Neurogenic Stress Cardiomyopathy After Aneurysmal Subarachnoid Hemorrhage. World Neurosurg..

[15] Abd T.T., Hayek S., Cheng J.W., Samuels O.B., Wittstein I.S., Lerakis S. (2014). Incidence and Clinical Characteristics of Takotsubo Cardiomyopathy Post-Aneurysmal Subarachnoid Hemorrhage. Int. J. Cardiol..

[16] Claassen J.A.H.R., Thijssen D.H.J., Panerai R.B., Faraci F.M. (2021). Regulation of Cerebral Blood Flow in Humans: Physiology and Clinical Implications of Autoregulation. Physiol. Rev..

[17] Havakuk O., King K.S., Grazette L., Yoon A.J., Fong M., Bregman N., Elkayam U., Kloner R.A. (2017). Heart Failure-Induced Brain Injury. J. Am. Coll. Cardiol..

[18] Gopinath R., Ayya S.S. (2018). Neurogenic Stress Cardiomyopathy: What Do We Need to Know. Ann. Card. Anaesth..

[19] Ziaka M., Exadaktylos A. (2023). The Heart Is at Risk: Understanding Stroke-Heart-Brain Interactions with Focus on Neurogenic Stress Cardiomyopathy-A Review. J. Stroke.

[20] Gyldenholm T., Hvas C.L., Hvas A.M., Hviid C.V.B. (2022). Serum Glial Fibrillary Acidic Protein (GFAP) Predicts Outcome after Intracerebral and Subarachnoid Hemorrhage. Neurol. Sci..

[21] Helbok R., Schiefecker A., Delazer M., Beer R., Bodner T., Pfausler B., Benke T., Lackner P., Fischer M., Sohm F. (2015). Cerebral Tau Is Elevated after Aneurysmal Subarachnoid Haemorrhage and Associated with Brain Metabolic Distress and Poor Functional and Cognitive Long-Term Outcome. J. Neurol. Neurosurg. Psychiatry.

[22] Kang C., You Y., Ahn H.J., Park J.S., Jeong W., Min J.H., In Y.N., Yoo I., Cho Y., Ryu S. (2022). Blood–Brain Barrier Disruption as a Cause of Various Serum Neuron-Specific Enolase Cut-off Values for Neurological Prognosis in Cardiac Arrest Patients. Sci. Rep..

[23] Balança B., Ritzenthaler T., Gobert F., Richet C., Bodonian C., Carrillon R., Terrie A., Desmurs L., Perret-Liaudet A., Dailler F. (2020). Significance and Diagnostic Accuracy of Early S100b Serum Concentration after Aneurysmal Subarachnoid Hemorrhage. J. Clin. Med..

[24] Jacob R., Khan M. (2018). Cardiac Biomarkers: What Is and What Can Be. Indian. J. Cardiovasc. Dis. Women WINCARS.

[25] Budohoski K.P., Czosnyka M., Smielewski P., Varsos G.V., Kasprowicz M., Brady K.M., Pickard J.D., Kirkpatrick P.J. (2016). Monitoring Cerebral Autoregulation after Subarachnoid Hemorrhage. Acta Neurochir. Suppl..

[26] Sykora M., Diedler J., Rupp A., Turcani P., Rocco A., Steiner T. (2008). Impaired Baroreflex Sensitivity Predicts Outcome of Acute Intracerebral Hemorrhage. Crit. Care Med..

[27] Szabo J., Smielewski P., Czosnyka M., Jakubicek S., Krebs S., Siarnik P., Sykora M. (2018). Heart Rate Variability Is Associated with Outcome in Spontaneous Intracerebral Hemorrhage. J. Crit. Care.

[28] Uryga A., Burzyńska M., Tabakow P., Kasprowicz M., Budohoski K.P., Kazimierska A., Smielewski P., Czosnyka M., Goździk W. (2018). Baroreflex Sensitivity and Heart Rate Variability Are Predictors of Mortality in Patients with Aneurysmal Subarachnoid Haemorrhage. J. Neurol. Sci..

[29] (1988). Report of World Federation of Neurological Surgeons Committee on a Universal Subarachnoid Hemorrhage Grading Scale. J. Neurosurg..

[30] Fisher C.M., Kistler J.P., Davis J.M. (1980). Relation of Cerebral Vasospasm to Subarachnoid Hemorrhage Visualized by Computerized Tomographic Scanning. Neurosurgery.

[31] Hijdra A., Brouwers P., Vermeulen M., Gijn J. (1990). Van Grading the Amount of Blood on Computed Tomograms after Subarachnoid Hemorrhage. Stroke.

[32] Ahn S.H., Savarraj J.P., Pervez M., Jones W., Park J., Jeon S.B., Kwon S.U., Chang T.R., Lee K., Kim D.H. (2018). The Subarachnoid Hemorrhage Early Brain Edema Score Predicts Delayed Cerebral Ischemia and Clinical Outcomes. Neurosurgery.

[33] Connolly E.S., Rabinstein A.A., Carhuapoma J.R., Derdeyn C.P., Dion J., Higashida R.T., Hoh B.L., Kirkness C.J., Naidech A.M., Ogilvy C.S. (2012). Guidelines for the Management of Aneurysmal Subarachnoid Hemorrhage: A Guideline for Healthcare Professionals from the American Heart Association/American Stroke Association. Stroke.

[34] Vergouwen M.D.I., Vermeulen M., van Gijn J., Rinkel G.J.E., Wijdicks E.F., Muizelaar J.P., Mendelow A.D., Juvela S., Yonas H., Terbrugge K.G. (2010). Definition of Delayed Cerebral Ischemia after Aneurysmal Subarachnoid Hemorrhage as an Outcome Event in Clinical Trials and Observational Studies: Proposal of a Multidisciplinary Research Group. Stroke.

[35] Davison D.L., Terek M., Chawla L.S. (2012). Neurogenic Pulmonary Edema. Crit. Care.

[36] Czosnyka M., Smielewski P., Kirkpatrick P., Piechnik S., Laing R., Pickard J.D. (1998). Continuous Monitoring of Cerebrovascular Pressure-Reactivity in Head Injury. Acta Neurochir. Suppl..

[37] Czosnyka M., Smielewski P., Kirkpatrick P., Menon D.K., Pickard J.D. (1996). Monitoring of Cerebral Autoregulation in Head-Injured Patients. Stroke.

[38] Westerhof B.E., Gisolf J., Stok W.J., Wesseling K.H., Karemaker J.M. (2004). Time-Domain Cross-Correlation Baroreflex Sensitivity: Performance on the EUROBAVAR Data Set. J. Hypertens..

[39] Malik M., Bigger J., Camm A., Kleiger R. (1996). Heart Rate Variability. Standards of Measurement, Physiological Interpretation, and Clinical Use. Task Force of the European Society of Cardiology and the North American Society of Pacing and Electrophysiology. Eur. Heart J..

[40] Quinn T.J., Dawson J., Walters M.R., Lees K.R. (2009). Reliability of the Modified Rankin Scale: A Systematic Review. Stroke.

[41] McAteer A., Hravnak M., Chang Y., Crago E.A., Gallek M.J., Yousef K.M. (2017). The Relationships Between BNP and Neurocardiac Injury Severity, Noninvasive Cardiac Output, and Outcomes After Aneurysmal Subarachnoid Hemorrhage. Biol. Res. Nurs..

[42] Chen Y., Cai C., Fei J., Luo S., You C. (2022). The Elevation of Different Myocardial Biomarkers on Admission Is Associated with Disease Features and Different Outcomes in Aneurysmal Subarachnoid Hemorrhage. Sci. Rep..

[43] Zahid T., Eskander N., Emamy M., Ryad R., Jahan N. (2020). Cardiac Troponin Elevation and Outcome in Subarachnoid Hemorrhage. Cureus.

[44] Temes R.E., Tessitore E., Schmidt J.M., Naidech A.M., Fernandez A., Ostapkovich N.D., Frontera J.A., Wartenberg K.E., Di Tullio M.R., Badjatia N. (2010). Left Ventricular Dysfunction and Cerebral Infarction from Vasospasm after Subarachnoid Hemorrhage. Neurocrit. Care.

[45] Kaculini C., Sy C., Lacci J.V., Jafari A.A., Mirmoeeni S., Seifi A. (2022). The Association of Takotsubo Cardiomyopathy and Aneurysmal Subarachnoid Hemorrhage: A U.S. Nationwide Analysis. Clin. Neurol. Neurosurg..

[46] Redfors B., Vedad R., Angerås O., Råmunddal T., Petursson P., Haraldsson I., Ali A., Dworeck C., Odenstedt J., Ioaness D. (2015). Mortality in Takotsubo Syndrome Is Similar to Mortality in Myocardial Infarction—A Report from the SWEDEHEART Registry. Int. J. Cardiol..

[47] Wang X.-C., Gao S.-J., Zhuo S.-L., Weng C.-L., Feng H.-W., Lin J., Lin X.-S., Huang L. (2023). Predictive Factors for Cerebrocardiac Syndrome in Patients with Severe Traumatic Brain Injury: A Retrospective Cohort Study. Front. Neurol..

[48] Chen Z., Venkat P., Seyfried D., Chopp M., Yan T., Chen J. (2017). Brain-Heart Interaction: Cardiac Complications After Stroke. Circ. Res..

[49] Scheitz J.F., Sposato L.A., Schulz-Menger J., Nolte C.H., Backs J., Endres M. (2022). Stroke-Heart Syndrome: Recent Advances and Challenges. J. Am. Heart Assoc..

[50] Sposato L.A., Hilz M.J., Aspberg S., Murthy S.B., Bahit M.C., Hsieh C.Y., Sheppard M.N., Scheitz J.F. (2020). Post-Stroke Cardiovascular Complications and Neurogenic Cardiac Injury: JACC State-of-the-Art Review. J. Am. Coll. Cardiol..

[51] Marenholz I., Heizmann C.W., Fritz G. (2004). S100 Proteins in Mouse and Man: From Evolution to Function and Pathology (Including an Update of the Nomenclature). Biochem. Biophys. Res. Commun..

[52] Herrmann M., Vos P., Wunderlich M.T., De Bruijn C.H.M.M., Lamers K.J.B. (2000). Release of Glial Tissue-Specific Proteins after Acute Stroke: A Comparative Analysis of Serum Concentrations of Protein S-100B and Glial Fibrillary Acidic Protein. Stroke.

[53] Janigro D., Mondello S., Posti J.P., Unden J. (2022). GFAP and S100B: What You Always Wanted to Know and Never Dared to Ask. Front. Neurol..

[54] Guzel A., Er U., Tatli M., Aluclu U., Ozkan U., Duzenli Y., Satici O., Guzel E., Kemaloglu S., Ceviz A. (2008). Serum Neuron-Specific Enolase as a Predictor of Short-Term Outcome and Its Correlation with Glasgow Coma Scale in Traumatic Brain Injury. Neurosurg. Rev..

[55] Zaheer S., Beg M., Rizvi I., Islam N., Ullah E., Akhtar N. (2013). Correlation between Serum Neuron Specific Enolase and Functional Neurological Outcome in Patients of Acute Ischemic Stroke. Ann. Ind. Acad. Neurol..

[56] Correia M., Silva I., Gabriel D., Simrén J., Carneiro A., Ribeiro S., Dória H.M., Varela R., Aires A., Minta K. (2022). Early Plasma Biomarker Dynamic Profiles Are Associated with Acute Ischemic Stroke Outcomes. Eur. J. Neurol..

[57] Dias A., Silva L., Moura J., Gabriel D., Maia L.F. (2022). Fluid Biomarkers in Stroke: From Animal Models to Clinical Care. Acta Neurol. Scand..

[58] Stammet P. (2017). Blood Biomarkers of Hypoxic-Ischemic Brain Injury after Cardiac Arrest. Semin. Neurol..

[59] Larsson I.M., Wallin E., Kristofferzon M.L., Niessner M., Zetterberg H., Rubertsson S. (2014). Post-Cardiac Arrest Serum Levels of Glial Fibrillary Acidic Protein for Predicting Neurological Outcome. Resuscitation.

[60] Hanin A., Denis J.A., Frazzini V., Cousyn L., Imbert-Bismut F., Rucheton B., Bonnefont-Rousselot D., Marois C., Lambrecq V., Demeret S. (2022). Neuron Specific Enolase, S100-Beta Protein and Progranulin as Diagnostic Biomarkers of Status Epilepticus. J. Neurol..

[61] Meric E., Gunduz A., Turedi S., Cakir E., Yandi M. (2010). The Prognostic Value of Neuron-Specific Enolase in Head Trauma Patients. J. Emerg. Med..

[62] Meynaar I.A., Oudemans-Van Straaten H.M., Wetering J., Verlooy P., Slaats E.H., Bosman R.J., Spoel J.I., Zandstra D.F. (2003). Serum Neuron-Specific Enolase Predicts Outcome in Post-Anoxic Coma: A Prospective Cohort Study. Intensive Care Med..

[63] Wunderlich M.T., Lins H., Skalej M., Wallesch C.W., Goertler M. (2006). Neuron-Specific Enolase and Tau Protein as Neurobiochemical Markers of Neuronal Damage Are Related to Early Clinical Course and Long-Term Outcome in Acute Ischemic Stroke. Clin. Neurol. Neurosurg..

[64] Abboud T., Mende K.C., Jung R., Czorlich P., Vettorazzi E., Priefler M., Kluge S., Westphal M., Regelsberger J. (2017). Prognostic Value of Early S100 Calcium Binding Protein B and Neuron-Specific Enolase in Patients with Poor-Grade Aneurysmal Subarachnoid Hemorrhage: A Pilot Study. World Neurosurg..

[65] Moritz S., Warnat J., Bele S., Graf B.M., Woertgen C. (2010). The Prognostic Value of NSE and S100B from Serum and Cerebrospinal Fluid in Patients with Spontaneous Subarachnoid Hemorrhage. J. Neurosurg. Anesthesiol..

[66] Tawk R.G., Grewal S.S., Heckman M.G., Rawal B., Miller D.A., Edmonston D., Ferguson J.L., Navarro R., Ng L., Brown B.L. (2016). The Relationship Between Serum Neuron-Specific Enolase Levels and Severity of Bleeding and Functional Outcomes in Patients With Nontraumatic Subarachnoid Hemorrhage. Neurosurgery.

[67] Isgrò M.A., Bottoni P., Scatena R. (2015). Neuron-Specific Enolase as a Biomarker: Biochemical and Clinical Aspects. Adv. Exp. Med. Biol..

[68] Kosik K.S., Finch E.A. (1987). MAP2 and Tau Segregate into Dendritic and Axonal Domains after the Elaboration of Morphologically Distinct Neurites: An Immunocytochemical Study of Cultured Rat Cerebrum. J. Neurosci..

[69] Schiefecker A.J., Dietmann A., Beer R., Pfausler B., Lackner P., Kofler M., Fischer M., Broessner G., Sohm F., Mulino M. (2017). Neuroinflammation Is Associated with Brain Extracellular TAU-Protein Release After Spontaneous Subarachnoid Hemorrhage. Curr. Drug Targets.

[70] Heilig M., Rass V., Lindner A., Kofler M., Ianosi B.A., Gaasch M., Putnina L., Humpel C., Scherfler C., Zamarian L. (2023). Brain Microdialysate Tau Dynamics Predict Functional and Neurocognitive Recovery after Poor-Grade Subarachnoid Haemorrhage. Brain Commun..

[71] Zanier E.R., Longhi L., Fiorini M., Cracco L., Bersano A., Zoerle T., Branca V., Monaco S., Stocchetti N. (2008). Increased Levels of CSF Heart-Type Fatty Acid-Binding Protein and Tau Protein after Aneurysmal Subarachnoid Hemorrhage. Acta Neurochir. Suppl..

[72] Mazzini G.S., Schaf D.V., Oliveira Á.R., Gonçalves C.A., Belló-Klein A., Bordignon S., Bruch R.S., Campos G.F., Vassallo D.V., Souza D.O. (2005). The Ischemic Rat Heart Releases S100B. Life Sci..

[73] Snyder-Ramos S.A., Gruhlke T., Bauer H., Bauer M., Luntz A.P., Motsch J., Martin E., Vahl C.F., Missler U., Wiesmann M. (2004). Cerebral and Extracerebral Release of Protein S100B in Cardiac Surgical Patients. Anaesthesia.

[74] Pfeifer R., Ferrari M., Börner A., Deufel T., Figulla H.R. (2008). Serum Concentration of NSE and S-100b during LVAD in Non-Resuscitated Patients. Resuscitation.

[75] Truter N., Malan L., Essop M.F. (2023). Glial Cell Activity in Cardiovascular Diseases and Risk of Acute Myocardial Infarction. Am. J. Physiol. Heart Circ. Physiol..

[76] Saand A.R., Yu F., Chen J., Chou S.H.Y. (2019). Systemic Inflammation in Hemorrhagic Strokes—A Novel Neurological Sign and Therapeutic Target?. J. Cereb. Blood Flow. Metab..

[77] Winek K., Soreq H., Meisel A. (2021). Regulators of Cholinergic Signaling in Disorders of the Central Nervous System. J. Neurochem..

[78] Bjerkne Wenneberg S., Löwhagen Hendén P.M., Oras J., Naredi S., Block L., Ljungqvist J., Odenstedt Hergès H. (2020). Heart Rate Variability Monitoring for the Detection of Delayed Cerebral Ischemia after Aneurysmal Subarachnoid Hemorrhage. Acta Anaesthesiol. Scand..

[79] Su I.C., Li C.H., Wang K.C., Lai D.M., Huang S.J., Shieh J.S., Tu Y.K. (2009). Prediction of Early Secondary Complications in Patients with Spontaneous Subarachnoid Hemorrhage Based on Accelerated Sympathovagal Ratios. Acta Neuroch..

[80] Chiu T.F., Huang C.C., Chen J.H., Chen W.L. (2012). Depressed Sympathovagal Balance Predicts Mortality in Patients with Subarachnoid Hemorrhage. Am. J. Emerg. Med..

[81] Burzyńska M., Uryga A., Kasprowicz M., Czosnyka M., Goździk W., Robba C. (2023). Cerebral Autoregulation, Cerebral Hemodynamics, and Injury Biomarkers, in Patients with COVID-19 Treated with Veno-Venous Extracorporeal Membrane Oxygenation. Neurocrit. Care.

[82] Randhawa P.K., Jaggi A.S. (2016). Unraveling the Role of Adenosine in Remote Ischemic Preconditioning-Induced Cardioprotection. Life Sci..

[83] English S.W., Fergusson D., Chassé M., Turgeon A.F., Lauzier F., Griesdale D., Algird A., Kramer A., Tinmouth A., Lum C. (2016). Aneurysmal SubArachnoid Hemorrhage—Red Blood Cell Transfusion And Outcome (SAHaRA): A Pilot Randomised Controlled Trial Protocol. BMJ Open.

[84] Al-Khindi T., MacDonald R.L., Schweizer T.A. (2010). Cognitive and Functional Outcome after Aneurysmal Subarachnoid Hemorrhage. Stroke.

[85] Passier P.E.C.A., Visser-Meily J.M.A., Van Zandvoort M.J.E., Post M.W.M., Rinkel G.J.E., Van Heugten C. (2010). Prevalence and Determinants of Cognitive Complaints after Aneurysmal Subarachnoid Hemorrhage. Cerebrovasc. Dis..

[86] Nobels-Janssen E., Abma I.L., Verhagen W.I.M., Bartels R.H.M.A., van der Wees P.J., Boogaarts J.D. (2021). Development of a Patient-Reported Outcome Measure for Patients Who Have Recovered from a Subarachnoid Hemorrhage: The “Questionnaire for the Screening of Symptoms in Aneurysmal Subarachnoid Hemorrhage” (SOS-SAH). BMC Neurol..

[87] Winek K., Lobentanzer S., Nadorp B., Dubnov S., Dames C., Jagdmann S., Moshitzky G., Hotter B., Meisel C., Greenberg D.S. (2020). Transfer RNA Fragments Replace MicroRNA Regulators of the Cholinergic Poststroke Immune Blockade. Proc. Natl. Acad. Sci. USA.

