# Supplementary material for: Cerebrospinal Fluid and Serum Biomarker Insights in Aneurysmal Subarachnoid Haemorrhage: Navigating the Brain–Heart Interrelationship for Improved Patient Outcomes
Source: Biomedicines. 2023 Oct 19;11(10):2835. doi: 10.3390/biomedicines11102835 (PMC10604203; doi:10.3390/biomedicines11102835)
Supplement: Supplementary file 1 [file biomedicines-11-02835-s001.zip › biomedicines-2632326-supplementary.pdf]

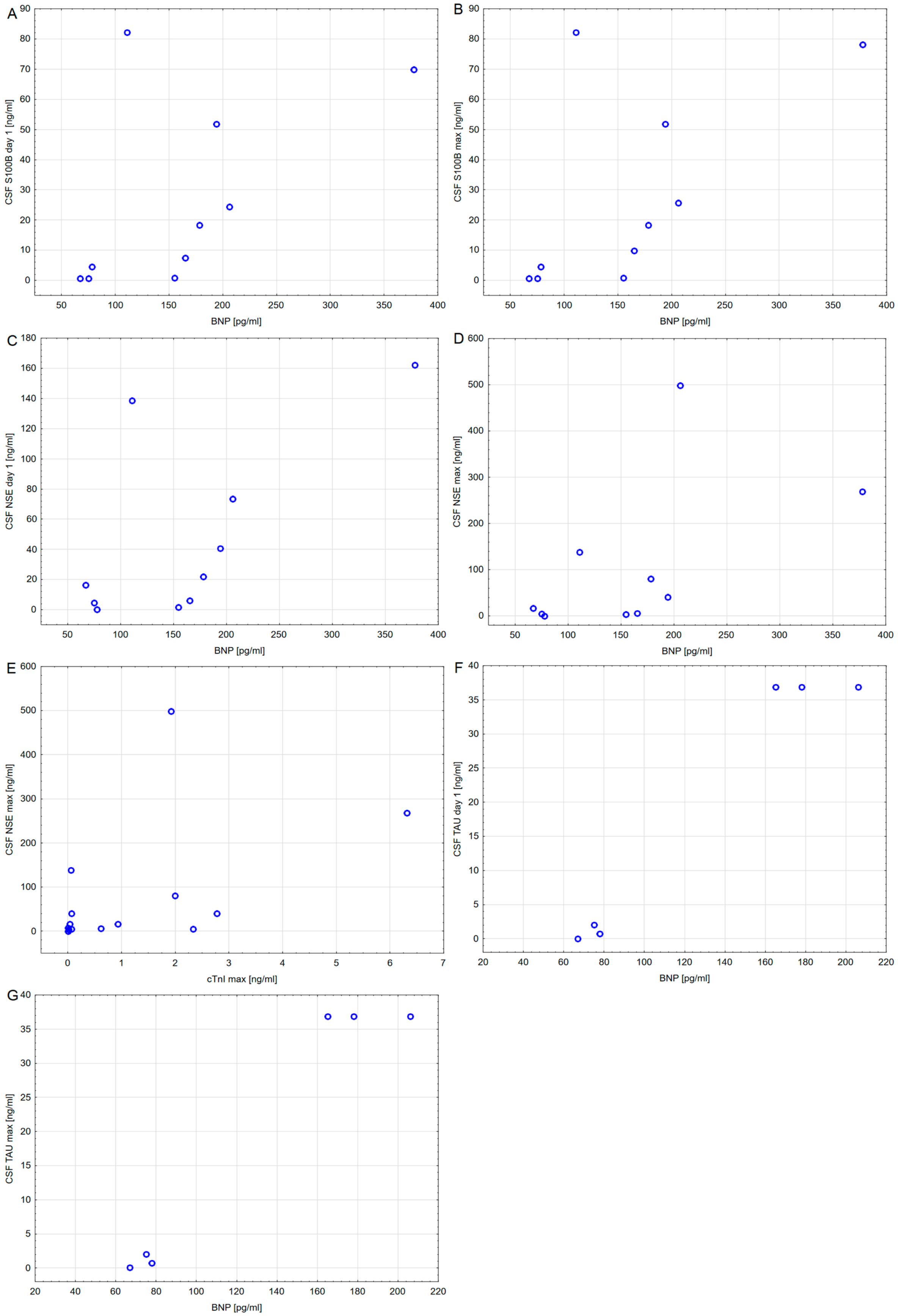

Supplementary Figure 1. Scatter plot presented correlation between: A) B-type Natriuretic Peptide (BNP) and cerebrospinal fluid (CSF) S100 Calcium-Binding Protein B (S100B) on day 1, B) BNP and maximal concentration from days 1-3 (max) of CSF S100B, C) BNP and CSF neuron-specific enolase (NSE) on day 1, D) BNP and CSF NSE max, E) cardiac troponine type I (cTnI) max and CSF NSE max, F) BNP and CSF TAU on day 1, G) BNP and CSF TAU max. BNP was evaluated in 11 patients; TAU was evaluated in 11 patients; other biomarkers were evaluated in 15 patients. CSF refers to biomarker levels measured in cerebrospinal fluid; if not indicated biomarker levels were measured in serum.

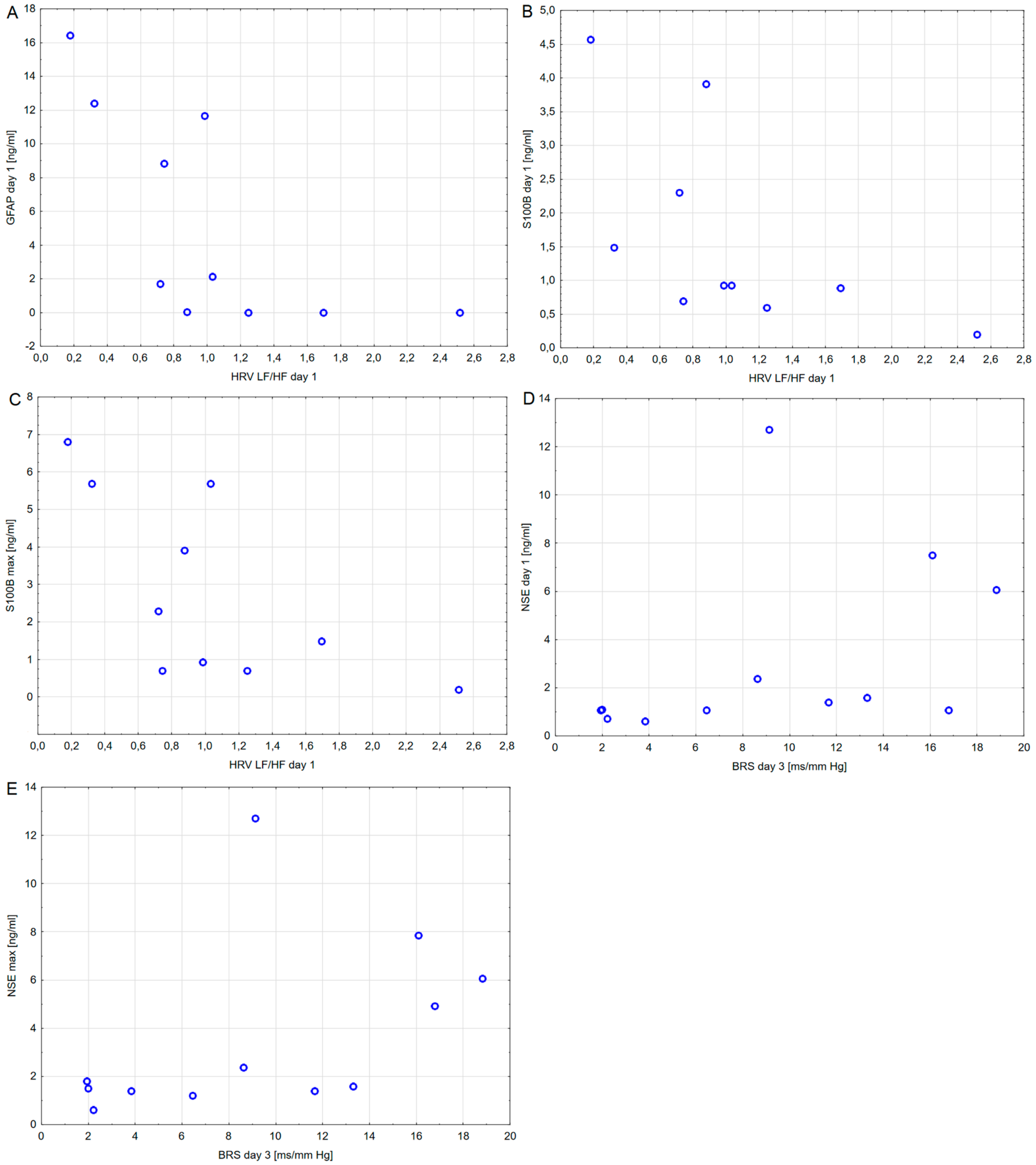

Supplementary Figure 2. The scatter plots presented a correlation between the autonomic nervous system (baroreflex sensitivity, BRS; heart rate variability as the ratio between low-frequency range (LF, 0.04-0.15 Hz) and high-frequency range (HF, 0.15-0.40 Hz), HRV LF/HF) and biomarkers: glial fibrillary acidic protein (GFAP); neuron-specific enolase (NSE); S100 calcium-binding protein (S100B); if not indicated otherwise biomarker levels were measured in serum.

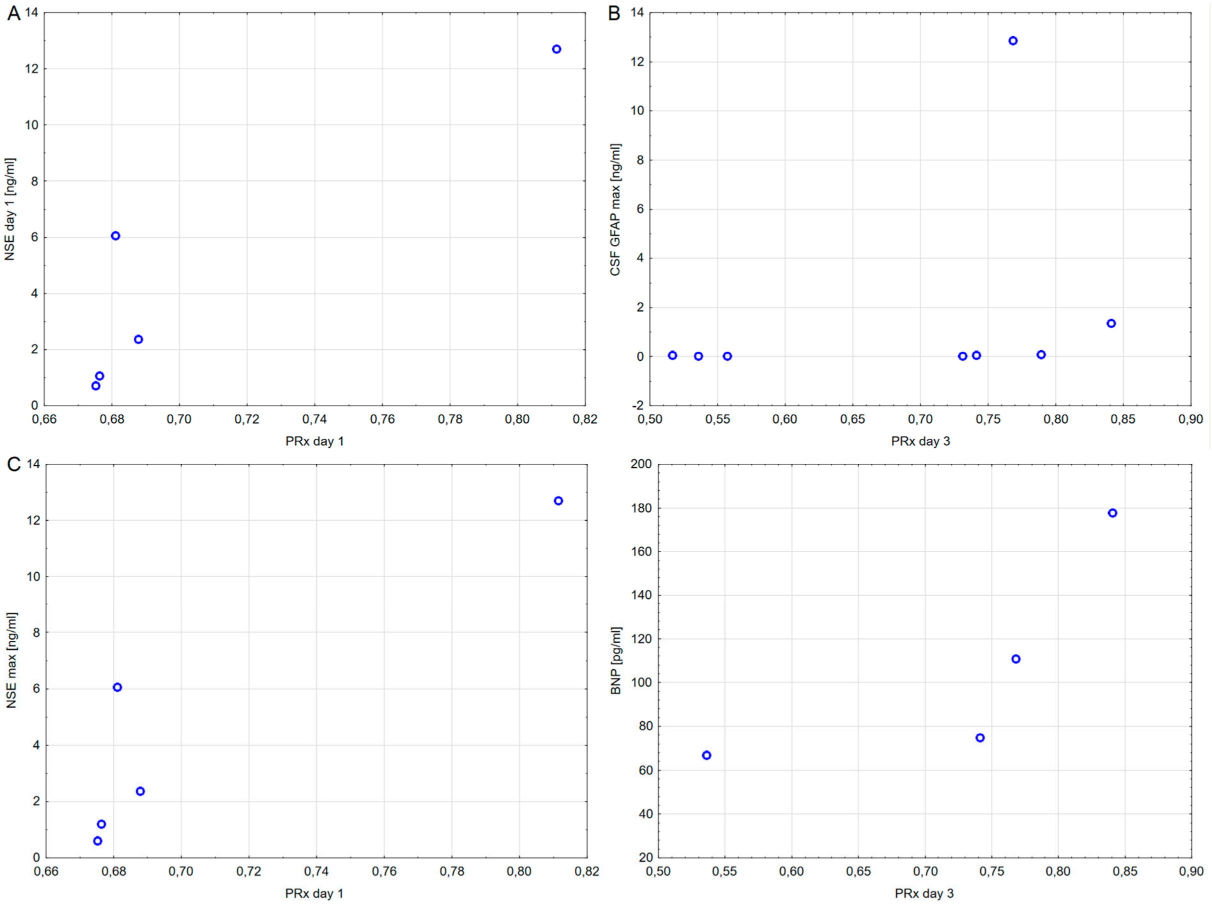

Supplementary Figure 3. The scatter plots presented a correlation between the cerebral autoregulation (pressure reactivity index, PRx) and biomarkers: glial fibrillary acidic protein (GFAP); neuron-specific enolase (NSE); B-type Natriuretic Peptide (BNP); CSF refers to biomarker levels measured in cerebrospinal fluid; if not indicated biomarker levels were measured in serum. PRx were monitored in 9 patients. BNP was evaluated in 11 patients.
